# Expression of embryonal stem cell transcription factors in breast cancer: Oct4 as an indicator for poor clinical outcome and tamoxifen resistance

**DOI:** 10.18632/oncotarget.16750

**Published:** 2017-03-31

**Authors:** Jae Moon Gwak, Milim Kim, Hyun Jeong Kim, Min Hye Jang, So Yeon Park

**Affiliations:** ^1^ Department of Pathology, Seoul National University College of Medicine, Seoul, Republic of Korea; ^2^ Green Cross Laboratory, Yongin, Republic of Korea; ^3^ Department of Pathology, Seoul National University Bundang Hospital, Seongnam, Republic of Korea; ^4^ Department of Pathology, Yeungnam University Medical Center, Daegu, Republic of Korea

**Keywords:** Oct4, breast cancer, cancer stem cell, prognosis, tamoxifen resistance

## Abstract

The transcription factors of embryonic stem cells, such as Oct4, Sox2, Nanog, Bmi1, and Klf4, are known to be associated with stemness, epithelial–mesenchymal transition and aggressive tumor behavior. This study was designed to evaluate the clinicopathological significance of their expression in breast cancer. Immunohistochemistry for Oct4, Sox2, Nanog, Bmi1, and Klf4 was performed in 319 cases of invasive breast cancer. The relationship between the expression of these markers and clinicopathologic features of the tumors, including breast cancer stem cell phenotype and epithelial–mesenchymal transition marker expression, and their prognostic value in breast cancer, were analyzed. Expression of Oct4 and Sox2 was commonly associated with high histologic grade and high Ki-67 index in the whole group and in the hormone receptor-positive subgroup. On the other hand, expression of Nanog, Bmi1, and Klf4 was inversely correlated with aggressive features of the breast cancer. Oct4 expression was associated with ALDH1 expression but not with epithelial–mesenchymal transition marker expression. In survival analysis, Oct4 expression was independently associated with poor prognosis in the whole group and in the hormone receptor-positive subgroup, but not in hormone receptor-negative subgroup. Particularly, Oct4 expression was associated with poor clinical outcome in patients with hormone receptor-positive breast cancer treated with tamoxifen. Our results indicate that Oct4 expression is associated with aggressive features, ALDH1 expression, tamoxifen resistance and poor clinical outcomes in hormone receptor-positive breast cancer, and thus may be useful as a predictive and prognostic marker in this subgroup of breast cancer.

## INTRODUCTION

Cancer stem cells (CSC) are regarded as a subpopulation of tumor cells that have ‘stem-like’ properties and the ability to sustain tumorigenesis [1, 2]. In other words, CSCs share some of fundamental features of normal stem cells, such as self-renewal and differentiation capacity, unlike other tumor cell lineages [3]. In breast cancer, Al-Hajj et al. demonstrated that only CD44^+^CD24^−/low^ lineage^−^ cells could form new tumors [4]. Aldehyde dehydrogenase (ALDH) activity was also found to be increased in breast CSCs (BCSCs) [5]. Epithelial–mesenchymal transition (EMT) is known to be closely associated with CSCs. Inducing EMT in immortalized human mammary epithelial cells resulted in increased ability to form mammospheres and the expression of the stem cell markers [6]. Therefore, identifying the CSC population and the markers they express may be important for predicting tumor progression and developing agents for targeted therapy.

In the developmental stage, the embryo has pluripotent stem cells called embryonic stem cells (ESCs), derived from the inner cell mass of blastocysts. They have the capability to replicate indefinitely while retaining the ability to differentiate into functionally distinct cell types [7]. The transcription factors of ESCs include octamer-binding transcription factor 4 (Oct4), sex determining region Y-box 2 (Sox2), Nanog, B cell-specific Moloney murine leukemia virus integration site 1 (Bmi1), and kruppel-like factor 4 (Klf4). These transcription factors are thought to be involved in the regulatory circuitry of ESCs, and to contribute to tumorigenesis and the progression of human breast cancer [8–12]. Wang et al. showed that overexpression of Oct4 and Nanog enhanced spontaneous changes in the expression of EMT-related genes in CSCs, and promoted the invasiveness of CSCs, and they suggested that Oct4 and Nanog could serve as markers of poor prognosis [10]. Other workers showed that increased Sox2 expression was related to adverse breast carcinoma profile, less differentiated subtype and poor outcomes in patients with high nodal stages [9]. Paranjape et al. found that Bmi1 was overexpressed in high-grade invasive ductal carcinoma, and that it increased the self-renewal activity of tumor cells, and also promoted EMT. They knocked out the Bmi1 gene and observed reversal of the EMT and reduced stemness [11]. Klf4 is also reported to be highly expressed in CSC-enriched populations, and to promote stem cell-like features, cell migration and invasion [12].

As mentioned above, ESC transcription factors have been shown to be associated with stemness, EMT and aggressive tumor activity. However, the overall relationships between these markers and breast cancer characteristics are not fully understood. We designed this study to evaluate the expression of ESC transcription factors (Oct4, Sox2, Nanog, Bmi1, and Klf4) in human invasive breast cancer samples, and to analyze their association with the clinicopathologic features of tumors including BCSC phenotype, EMT marker expression, molecular subtype, and prognosis.

## RESULTS

### Patient characteristics

Median age of patients was 50.9 years (range, 26–87). The size of tumor was 2cm or less (pT1) in 51.1%. Lymph node metastasis was detected in 136 (42.6%) cases. Of all cases, 221 (69.3%) were positive for estrogen receptor (ER). *HER2* amplification was identified in 81 (25.4%) cases. The rest of baseline characteristics are listed in Table 1.

**Table 1:** Baseline characteristics

Characteristic	Number (%)
Age, yrs.	
Median	50.9
Range	26–87
T stage	
T1	163 (51.1)
T2	148 (46.4)
T3	5 (1.6)
T4	3 (0.9)
N stage	
N0	183 (57.4)
N1	91 (28.5)
N2	24 (7.5)
N3	21 (6.6)
Histologic subtype	
No special type	288 (90.3)
Lobular	6 (1.9)
Micropapillary	5 (1.6)
Metaplastic	9 (2.8)
Mucinous	7 (2.2)
Others	4 (1.3)
Histologic grade	
I	45 (14.1)
II	94 (29.5)
III	180 (56.4)
LVI	
Absent	173 (54.2)
Present	146 (45.8)
P53 overexpression	
Negative	221 (69.3)
Positive	98 (30.7)
Ki-67	
< 20%	166 (52.0)
≥ 20%	153 (48.0)
ER	
Negative	98 (30.7)
Positive	221 (69.3)
PR	
Negative	145 (45.5)
Positive	174 (54.5)
HER2	
Negative	238 (74.6)
Positive	81 (25.4)
Subtype	
Luminal A	119 (37.3)
Luminal B	104 (32.6)
HER2+	40 (12.5)
Triple-negative	56 (17.6)
Adjuvant chemotherapy	
Not received	48 (15.0)
Received	265 (83.1)
Unknown	6 (1.9)
Adjuvant radiotherapy	
Not received	137 (42.9)
Received	176 (55.2)
Unknown	6 (1.9)
Adjuvant hormone therapy	
Not received	106 (33.2)
Received	207 (64.9)
Unknown	6 (1.9)

LVI, lymphovascular invasion; ER, estrogen receptor; PR, progesterone receptor; HER2, human epidermal growth factor receptor 2

### Expression of ESC transcription factors in relation to clinicopathologic features of the tumors

**Figure 1:**
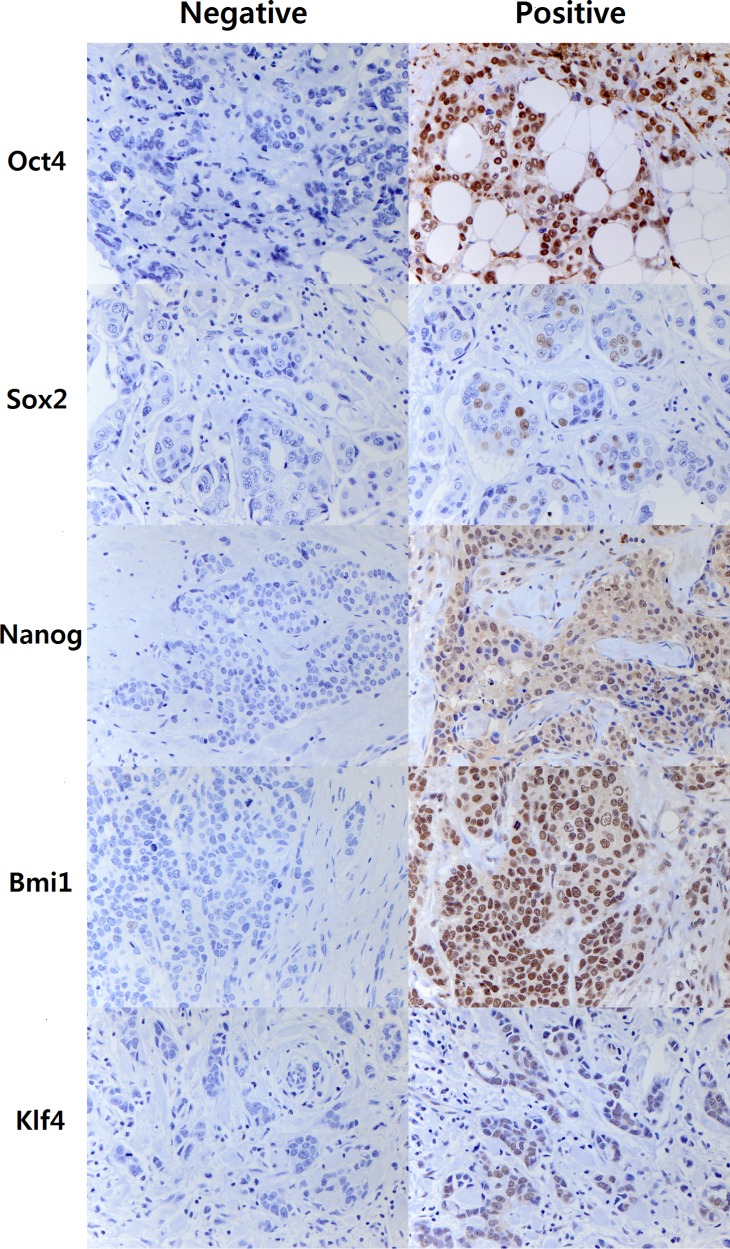
Representative images of immunohistochemal staining of embryonal stem cell transcription factors Nuclear staining in 10% or more of the tumor cells is considered as positive for Oct4, Nanog, Bmi1 and Klf4, while nuclear staining in 1% or more of tumor cells is regarded as positive for Sox2 due to the rarity in its expression. Right column represents positive staining of Oct4, Sox2, Nanog, Bmi1, and Klf4 in breast cancer tissues (Original magnification: ×400).

Oct4, Sox2, Nanog, Bmi1, and Klf4 were expressed in 15.4%, 10.3%, 20.4%, 50.5%, and 17.6% of tumor samples, respectively (Figure 1). Oct4 and Sox2 expression was higher in tumors of high histologic grade and high Ki-67 proliferation index (all *p* < 0.05). Moreover, Oct4 expression was associated with *HER2* amplification (*p* = 0.007) and negative ER status (*p* = 0.019). Sox2 expression was marginally associated with p53 overexpression (*p* = 0.053). On the other hand, expression of Nanog, Bmi1, and Klf4 was inversely correlated with aggressive features of the breast cancers. Their expression was more frequent in hormone receptor-positive breast cancers and in tumor with low histologic grade (all *p* < 0.05). In addition, Bmi1 expression was higher in tumors of low Ki-67 proliferation index and in which p53 was not overexpressed (all *p* < 0.05). Klf4 was also associated with absence of p53 overexpression (*p* = 0.048). Nanog expression was associated with nodal metastasis (*p* = 0.009). The relationships between clinicopathologic variables and expression of ESC transcription factors are summarized in Table 2 and Supplementary Table 1.

**Table 2:** Relationship between clinicopathologic characteristics of tumors and expression of Oct4 and Sox2 assessed by immunohistochemistry

Clinicopathologic Characteristic	Number	Oct4		p value	Sox2		p value
		Negative N (%)	Positive N (%)		Negative N(%)	Positive N (%)	
T stage				0.991			0.155
T1	163	138 (51.1)	25 (51.0)		150 (52.4)	13 (39.4)	
T2–T4	156	132 (48.9)	24 (49.0)		136 (47.6)	20 (60.6)	
N stage				0.222			0.729
N0	183	151 (55.9)	32 (65.3)		165 (57.7)	18 (54.5)	
N1–N3	136	119 (44.1)	17 (34.7)		121 (42.3)	15 (45.5)	
Histologic grade				0.030			0.019
I	45	44 (16.3)	1 (2.0)		45 (15.7)	0 (0)	
II	94	78 (28.9)	16 (32.7)		86 (30.1)	8 (24.2)	
III	180	148 (54.8)	32 (65.3)		155 (54.2)	25 (75.8)	
LVI				0.624			0.970
Absent	173	148 (54.8)	25 (51.0)		155 (54.2)	18 (54.5)	
Present	146	122 (45.2)	24 (49.0)		131 (45.8)	15 (45.5)	
P53 overexpression				0.321			0.053
Negative	221	190 (70.4)	31 (63.3)		203 (71.0)	18 (54.5)	
Positive	98	80 (29.6)	18 (36.7)		83 (29.0)	15 (45.5)	
Ki-67				< 0.001			< 0.001
< 20%	142	153 (56.7)	14 (28.6)		160 (55.9)	7 (21.2)	
≥ 20%	177	117 (43.3)	35 (71.4)		126 (44.1)	26 (78.8)	
ER				0.019			0.254
Negative	98	76 (28.1)	22 (44.9)		85 (29.7)	13 (39.4)	
Positive	221	194 (71.9)	27 (55.1)		201 (70.3)	20 (60.6)	
PR				0.245			0.712
Negative	145	119 (44.1)	26 (53.1)		129 (45.1)	16 (48.5)	
Positive	174	151 (55.9)	23 (46.9)		157 (54.9)	17 (51.5)	
HER2				0.007			0.268
Negative	238	209 (77.4)	29 (59.2)		216 (75.5)	22 (66.7)	
Positive	81	61 (22.6)	20 (40.8)		70 (24.5)	11 (33.3)	

*P* values were calculated by chi-square test or Fisher's exact test.

LVI, lymphovascular invasion; ER, estrogen receptor; PR, progesterone receptor; HER2, human epidermal growth factor receptor 2.

### Expression of ESC transcription factors in relation to BCSC phenotype and EMT marker expression

In the next step, we examined the relationships between expression of ESC transcription factors and expression of BSCS and EMT markers (Table 3 and Supplementary Table 2). Oct4 expression was associated with ALDH1 expression (*p* < 0.001), while expression of Bmi1 and Klf4 was inversely correlated with ALDH1 expression (*p* = 0.049, *p* = 0.008, respectively).

**Table 3:** Expression of Oct4 and Sox2 in relation to BCSC phenotypes and EMT markers

Marker	Number	Oct4		p value	Sox2		p value
		Negative N (%)	Positive N (%)		Negative N (%)	Positive N (%)	
CD44(+) CD24(−)				0.877			0.666
<10%	166	141 (52.2)	25 (51.0)		150 (52.4)	16 (48.5)	
≥10%	153	129 (47.8)	24 (49.0)		136 (47.6)	17 (51.5)	
ALDH1				< 0.001			0.532
< 10%	289	252 (93.3)	37 (75.5)		260 (90.9)	29 (87.9)	
≥ 10%	30	18 (6.7)	12 (24.5)		26 (9.1)	4 (12.1)	
Vimentin				0.593			0.119
< 10%	272	229 (84.8)	43 (87.8)		247 (86.4)	25 (75.8)	
≥ 10%	47	41 (15.2)	6 (12.2)		39 (13.6)	8 (24.2)	
E-cadherin loss*				0.661			0.183
< 50%	226	192 (72.5)	34 (69.4)		199 (70.8)	27 (81.8)	
≥ 50%	88	73 (27.5)	15 (30.6)		82 (29.2)	6 (18.2)	

*P* values were calculated by chi-square test or Fisher's exact test.

ALDH1, aldehyde dehydrogenase 1.

* Invasive lobular carcinoma cases were excluded.

With regard to EMT, expression of Oct4 or Sox2 was not associated with EMT marker expression. Nanog and Klf4 expression was lower in cases showing loss of E-cadherin (*p* = 0.002 and *p* = 0.005, respectively), and Nanog expression was also lower in tumors expressing vimentin (*p* = 0.010).

### Expression of ESC transcription factors according to breast cancer molecular subtype

**Figure 2:**
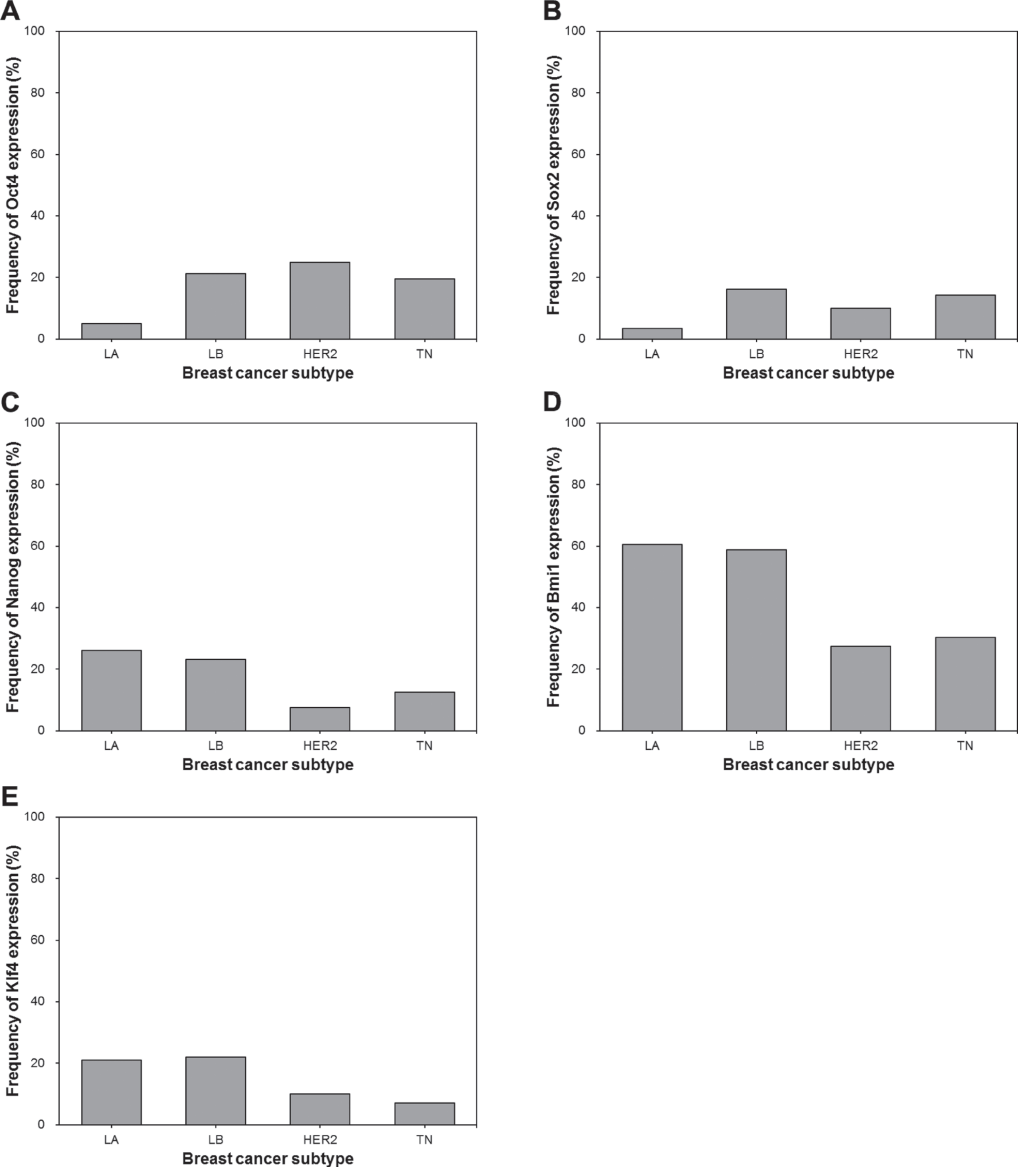
Frequency of Oct4, Sox2, Nanog, Bmi1 and Klf4 expression according to molecular subtype of breast cancer (**A**) The frequency of Oct4 expression is significantly lower in luminal A subtype than in luminal B, HER2+ and triple-negative subtypes (luminal A vs. luminal B, *p* < 0.001; luminal A vs. HER2+, *p* = 0.001. luminal A vs. triple-negative, *p* = 0.002). (**B**) Sox2 expression is less frequent in luminal A subtype than in luminal B and triple-negative subtypes (luminal A vs. luminal B, *p* = 0.001; luminal A vs. triple-negative, *p* = 0.020). (**C**) Nanog is more frequently expressed in luminal A and luminal B subtypes (luminal A vs. HER2+, *p* = 0.013; luminal A vs. triple-negative, *p* = 0.043; luminal B vs. HER2+, *p* = 0.032). (**D**) Bmi1 expression is more frequent in luminal A and luminal B subtypes than in HER2+ and triple-negative subtypes (luminal A vs. HER2+, *p* < 0.001; luminal A vs. triple-negative, *p* < 0.001; luminal B vs. HER2+, *p* = 0.001; luminal B vs. triple-negative, *p* = 0.001). (**E**) Klf4 is more frequently expressed in luminal A and luminal B subtypes than in triple-negative subtype (luminal A vs. triple-negative, *p* = 0.021; luminal B vs. triple-negative, *p* = 0.016). LA, luminal A; LB, luminal B; HER2, HER2+; TN, triple-negative.

We also examined the associations between expression of ESC transcription factors and the molecular subtypes of breast cancer. Expression of Oct4 and Sox2 was lowest in the luminal A subtype (Figure 2). On the other hand, Nanog, Bmi1, and Klf4 displayed a tendency to be highly expressed in the hormone receptor-positive subgroups (luminal A and luminal B). Specifically, Nanog expression was higher in the luminal A subtype than in the HER2+ or triple-negative subtypes and also higher in the luminal B than in the HER2+ subtype. Bmi1 was more highly expressed in the luminal A and B subtypes than in the HER2+ or triple-negative subtypes, while Klf4 expression was more common in the luminal A and B subtypes than in the triple-negative subtype (all *p* < 0.05).

### Analysis according to hormone receptor status

We also investigated the associations between ESC transcription factors and the clinicopathologic features of breast cancer according to hormone receptor status (Tables 4, 5 and Supplementary Tables 3, 4). First, in the hormone receptor-positive subgroup, Oct4 expression was associated with high histologic grade, high Ki-67 proliferation index, *HER2* amplification and ALDH1 expression, showing the same associations as in the whole group (all *p* < 0.05). Sox2 expression was positively correlated with histologic grade, Ki-67 proliferation index and p53 overexpression (all *p* < 0.05). Nanog expression was associated with nodal metastasis (*p* = 0.006) and E-cadherin retention (*p* = 0.012), as in the whole group. On the other hand, Bmi1 expression was not correlated with any clinicopathological features, and Klf4 expression only showed an inverse correlation with loss of E-cadherin (*p* = 0.047).

**Table 4:** Relationship between clinicopathologic characteristics of tumors and expression of Oct4 and Sox2 in the hormone receptor-positive subgroup

Clinicopathologic Characteristic	Number	Oct4		p value	Sox2		p value
		Negative N (%)	Positive N (%)		Negative N (%)	Positive N (%)	
T stage				0.703			0.311
T1	119	105 (53.8)	14 (50.0)		110 (54.5)	9 (42.9)	
T2-T4	104	90 (46.2)	14 (50.0)		92 (45.5)	12 (57.1)	
N stage				0.743			0.856
N0	121	105 (53.8)	16 (57.1)		110 (54.5)	11 (52.4)	
N1-N3	102	90 (46.2)	12 (42.9)		92 (45.5)	10 (47.6)	
Histologic grade				0.019			0.015
I	44	44 (22.6)	0 (0.0)		44 (21.8)	0 (0.0)	
II	86	72 (36.9)	14 (50.0)		78 (38.6)	8 (38.1)	
III	93	79 (40.5)	14 (50.0)		80 (39.6)	13 (61.9)	
LVI				0.164			0.938
Absent	115	104 (53.3)	11 (39.3)		104 (51.5)	11 (52.4)	
Present	108	91 (46.7)	17 (60.7)		98 (48.5)	10 (47.6)	
P53 overexpression				0.911			0.048
Negative	177	155 (79.5)	22 (78.6)		164 (81.2)	13 (61.9)	
Positive	46	40 (20.5)	6 (21.4)		38 (18.8)	8 (38.1)	
Ki-67				0.005			< 0.001
< 20%	155	142 (72.8)	13 (46.4)		149 (73.8)	6 (28.6)	
≥ 20%	68	53 (27.2)	15 (53.6)		53 (26.2)	15 (71.4)	
HER2				0.011			0.077
Negative	182	164 (84.1)	18 (64.3)		168 (83.2)	14 (66.7)	
Positive	41	31 (15.9)	10 (35.7)		34 (16.8)	7 (33.3)	
CD44(+)CD24(−)				0.492			0.725
< 10%	130	112 (57.4)	18 (64.3)		117 (57.9)	13 (61.9)	
≥ 10%	93	83 (42.6)	10 (35.7)		85 (42.1)	8 (38.1)	
ALDH1				0.044			0.505
< 10%	216	191 (97.9)	25 (89.3)		196 (97.0)	20 (95.2)	
≥ 10%	7	4 (2.1)	3 (10.7)		6 (3.0)	1 (4.8)	
Vimentin				0.380			1.000
< 10%	210	182 (93.3)	28 (100.0)		190 (94.1)	20 (95.2)	
≥10%	13	13 (6.7)	0 (0.0)		12 (5.9)	1 (4.8)	
E-cadherin loss*				0.588			0.171
< 50%	172	151 (79.5)	21 (75.0)		153 (77.7)	19 (90.5)	
≥ 50%	46	39 (20.5)	7 (25.0)		44 (22.3)	2 (9.5)	

*P* values were calculated by chi-square test or Fisher's exact test.

LVI, lymphovascular invasion; HER2, human epidermal growth factor receptor 2; ALDH1, aldehyde dehydrogenase 1

*Invasive lobular carcinoma cases were excluded.

**Table 5:** Relationship between clinicopathologic characteristics of tumors and expression of Oct4 and Sox2 in the hormone receptor-negative subgroup

Clinicopathologic Characteristic	Number	Oct4		p value	Sox2		p value
		Negative N (%)	Positive N (%)		Negative N (%)	Positive N (%)	
T stage				0.496			0.353
T1	44	33 (44.0)	11 (52.4)		40 (47.6)	4 (33.3)	
T2-T4	52	42 (56.0)	10 (47.6)		44 (52.4)	8 (66.7)	
N stage				0.208			0.749
N0	62	46 (61.3)	16 (76.2)		55 (65.5)	7 (58.3)	
N1-N3	34	29 (38.7)	5 (23.8)		29 (34.5)	5 (41.7)	
Histologic grade				0.201			0.641
I	1	0 (0.0)	1 (4.8)		1 (1.2)	0 (0.0)	
II	8	6 (8.0)	2 (9.5)		8 (9.5)	0 (0.0)	
III	87	69 (92.0)	18 (85.7)		75 (89.3)	12 (100.0)	
LVI				0.508			1.000
Absent	58	44 (58.7)	14 (66.7)		51 (60.7)	7 (58.3)	
Present	38	31 (41.3)	7 (33.3)		33 (39.3)	5 (41.7)	
P53 overexpression				0.757			0.757
Negative	44	35 (46.7)	9 (42.9)		39 (46.4)	5 (41.7)	
Positive	52	40 (53.3)	12 (57.1)		45 (53.6)	7 (58.3)	
Ki-67				0.446			1.000
< 20%	11	10 (13.3)	1 (4.8)		10 (11.9)	1 (8.3)	
≥ 20%	85	65 (86.7)	20 (95.2)		74 (88.1)	11 (91.7)	
HER2				0.531			0.531
Negative	56	45 (60.0)	11 (52.4)		48 (57.1)	8 (66.7)	
Positive	40	30 (40.0)	10 (47.6)		36 (42.9)	4 (33.3)	
CD44(+)CD24(−)				0.655			0.526
< 10%	36	29 (38.7)	7 (33.3)		33 (39.3)	3 (25.0)	
≥ 10%	60	46 (61.3)	14 (66.7)		51 (60.7)	9 (75.0)	
ALDH1				0.022			1.000
< 10%	73	61 (81.3)	12 (57.1)		64 (76.2)	9 (75.0)	
≥ 10%	23	14 (18.7)	9 (42.9)		20 (23.8)	3 (25.0)	
Vimentin				0.458			0.107
< 10%	62	47 (62.7)	15 (71.4)		57 (67.9)	5 (41.7)	
≥ 10%	34	28 (37.3)	6 (28.6)		27 (32.1)	7 (58.3)	
E-cadherin loss				0.555			0.437
< 50%	54	41 (54.7)	13 (61.9)		46 (54.8)	8 (66.7)	
≥ 50%	42	34 (45.3)	8 (38.1)		38 (45.2)	4 (33.3)	

*P* values were calculated by chi-square test or Fisher's exact test.

LVI, lymphovascular invasion; HER2, human epidermal growth factor receptor 2; ALDH1, aldehyde dehydrogenase 1.

In the hormone receptor-negative subgroup, Oct4 expression was not correlated with any clinicopathologic features of breast cancer, but showed a positive correlation with ALDH1 expression (*p* = 0.022). Sox2, Bmi1 and Klf4 showed no association with any features. Nanog expression was related to non-CD44(+)CD24(−) phenotype (*p* = 0.037).

### Oct4 as an independent negative prognostic indicator in hormone receptor-positive breast cancer

**Figure 3:**
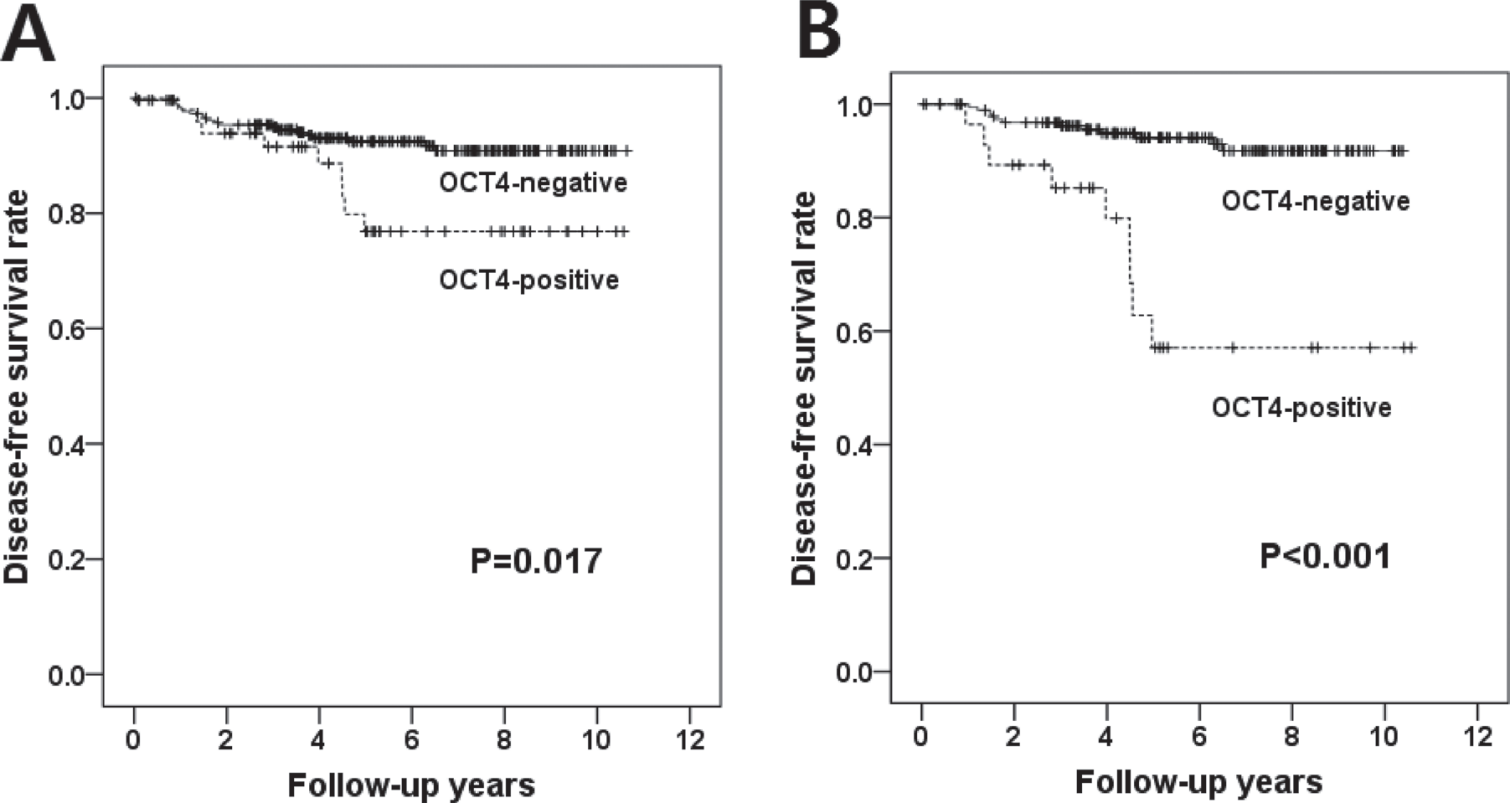
Disease-free survival according to Oct4 expression (**A**) In Kaplan-Meier survival analyses, patients with Oct4 expression have significantly shorter disease-free survival times than those without Oct4 expression in the whole patient group (*p* = 0.017, log-rank test). (**B**) Oct4 expression has more significant prognostic value in hormone receptor-positive subgroup (*p* < 0.001, log-rank test).

The median follow-up period for the 319 patients was 5.29 years (range, 0.04–10.64 years). During follow up, there were 29 tumor recurrences including 25 distant metastases and 4 local recurrences as first events. We performed Kaplan–Meier survival analysis to investigate the prognostic significance of all the clinicopathologic factors and the transcription factors of ESCs (Supplementary Table 5). Among the clinicopathologic features, nodal metastasis and lymphovascular invasion were associated with poor prognosis (*p* = 0.017 and *p* = 0.009, respectively). High T stage and high histologic grade also showed a tendency to be associated with poor disease-free survival (*p* = 0.139 and *p* = 0.075, respectively). Of the ESC transcription factors, only Oct4 expression was significantly correlated with shorter disease-free survival (*p* = 0.017; Figure 3A) and the expression status of the other ESC transcription factors was not related to survival. In multivariate analysis including T stage, N stage, histologic grade, lymphovascular invasion and Oct4 expression, nodal metastasis HR, 2.715; 95% CI, 1.254–5.875; *p* = 0.011) and Oct4 expression HR, 2.542; 95% CI, 1.144–5.647; *p* = 0.022) were found as independent factors for disease-free survival (Table 6).

**Table 6:** Univariate and multivariate analyses of disease-free survival

Variable	Category	Univariate analysis			Multivariate analysis		
		Hazard ratio	95% CI	p value	Hazard ratio	95% CI	p value
Whole group							
T stage	T1 vs. T2-4	1.750	0.826–3.706	0.144	1.219	0.561–2.653	0.617
N stage	N0 vs. N1-3	2.468	1.147–5.311	0.021	2.715	1.254–5.875	0.011
Histologic grade	I vs. II & III	5.099	0.694–37.488	0.109	4.247	0.570–31.626	0.158
LVI	Absent vs. Present	2.735	1.245–6.008	0.012	1.984	0.840–4.687	0.118
Oct4	Negative vs. Positive	2.522	1.148–5.542	0.021	2.542	1.144–5.647	0.022
Hormone receptor-positive group							
T stage	T1 vs. T2-4	2.546	1.027–6.309	0.044	2.067	0.827–5.168	0.120
N stage	N0 vs. N1-3	3.606	1.320–9.852	0.012	4.443	1.613–12.236	0.004
Histologic grade	I vs. II & III	5.533	0.742–41.246	0.095	2.789	0.352–22.089	0.331
LVI	Absent vs. Present	2.167	0.874–5.374	0.095	1.053	0.398–2.786	0.918
Oct4	Negative vs. Positive	6.346	2.662–15.128	<0.001	7.782	3.226–18.772	< 0.001
Hormone receptor-positive group treated with tamoxifen							
T stage	T1 vs. T2-4	2.029	0.772–5.334	0.151	1.755	0.658–4.678	0.261
N stage	N0 vs. N1-3	2.847	1.003–8.083	0.049	3.729	1.291–10.769	0.015
Histologic grade	I vs. II & III	3.246	0.430–24.504	0.254	1.434	0.175–11.720	0.737
LVI	Absent vs. Present	1.443	0.549–3.792	0.457	0.912	0.336–2.477	0.857
Oct4	Negative vs. Positive	5.662	2.179–14.716	<0.001	7.115	2.683–18.870	< 0.001

*P* values were calculated by Cox proportional hazards regression model with a backward stepwise selection method.

CI, confidence interval; LVI, lymphovascular invasion.

In the hormone receptor-positive subgroup, Oct4 expression was also related to poor disease-free survival (*p* < 0.001; Figure 3B). In multivariate analysis, nodal metastasis (HR, 4.443; 95% CI, 1.613–12.236; *p* = 0.004) and Oct4 expression (HR, 7.782; 95% CI, 3.226–18.772; *p <* 0.001) were revealed as independent negative prognostic factors (Table 6). A recent study showed that Oct4 acts on tamoxifen binding site and is involved in tamoxifen resistance [13]. Thus, we evaluated the relationship between Oct4 expression and tamoxifen resistance. Of the 207 patients receiving adjuvant hormone therapy, 129 patients were treated with tamoxifen. Oct4 expression was significantly associated with poor disease-free survival in hormone receptor-positive breast cancer patients treated with adjuvant tamoxifen in univariate and multivariate survival analyses (HR=5.662 [95% CI, 2.179–14.716], *p* < 0.001; HR=7.115 [95% CI, 2.683–18.870], *p* < 0.001; Table 6).

In the hormone receptor-negative subgroup, only lymphovascular invasion was associated with poor prognosis of patients (*p* = 0.033) and Oct4 expression did not show prognostic significance in this subgroup of breast cancer (*p* = 0.115).

## DISCUSSION

In this study, we enquired whether the transcription factors of ESCs are associated with tumor progression and BCSC or EMT marker expression in breast cancer. We demonstrated that Oct4 was highly expressed in breast cancers with aggressive features such as high histologic grade high Ki-67 proliferation index and *HER2* amplification, and the non-luminal A molecular subtypes. Its expression was more frequent in tumors expressing ALDH1, showing its association with the BCSC phenotype. The same associations were also found in the hormone receptor-positive subgroup. Finally, Oct4 was revealed as an independent negative prognostic factor in the whole group and in hormone receptor-positive subgroup, particularly in hormone receptor-positive subgroup treated with tamoxifen. To the best of our knowledge, this is the first study reporting the association of Oct4 expression with tamoxifen resistance and clinical outcome in hormone receptor-positive breast cancer using clinical samples.

Some previous studies already showed the prognostic significance of Oct4 expression in breast cancer [10, 14, 15]. However, most studies were confined to the small sized samples and some did not demonstrate prognostic significance of Oct4 as an independent factor. Moreover, those studies did not show the prognostic value of Oct4 expression according to hormone receptor status. In this study, we used a large set of breast cancer samples with complete clinical follow-up data and revealed that Oct4 expression is an independent poor prognostic factor in breast cancer. Furthermore, our study showed that prognostic value of Oct4 expression is more prominent in hormone receptor-positive breast cancer. Recently, Bhatt et al. [13] reported that Oct4 level was highly elevated in MCF-7-tam^r^ cells and was critical for their tamoxifen sensitivity. The relationship between Oct4 expression and patient prognosis in hormone receptor-positive group but not in hormone receptor-negative group may be associated with the action of Oct4 on tamoxifen resistance.

Oct4 is thought to play an important role in the EMT process. Knockout of Oct4 reduced the proliferation rate of a hepatocellular carcinoma cell line, and reversed EMT [16]. Co-expression of Oct4 and Nanog is assumed to promote EMT by activating Stat3/Snail signaling [17]. Chen et al. showed that Oct4 increased the invasiveness of lung cancer cells, and induced mesenchymal markers such as vimentin and N-cadherin. Oct4 also regulated degradation of the β-catenin/E-cadherin complex [18]. On the other hand, Hu et al. demonstrated that silencing Oct4 promoted the invasiveness and spread of breast cancer cell line MCF-7 by inducing EMT. This may imply a complex regulatory loop between Oct4 and EMT signals in breast cancer [19]. Moreover, Oct4/Sox2 overexpression was reported to decrease the expression of Snail, a key EMT inducer [20]. However, we detected no relationship between Oct4 expression and EMT marker expression. A dose-dependent effect of Oct4 could be one reason for this discrepancy because a precise level of Oct3/4 is needed to sustain maximum stemness or pluripotency [21].

Among the other transcription factors of ESCs evaluated in this study, Sox2 was positively related to tumor aggressiveness, along with Oct4. However, contrary to previous studies [9, 22], it was not associated with clinical outcome of the patients. This discrepancy may be associated with differences in sample platform, criteria for scoring and cutoff points for positive staining. The methodology for measuring Sox2 expression needs to be investigated since a recent meta-analysis reported that the cutoff points and standards for Sox2 immunochemistry differed in arbitrary fashion between studies [23]. Moreover, because Sox2 is expressed preferentially in the less-differentiated basal-like breast cancer subtypes, differences in the distribution of molecular subtypes between samples may alter outcomes [24]. Furthermore complex interactions of Sox2 with its partner proteins and its relatively low expression rate compared with other ESC transcription factors in breast cancer could lead to variable outcomes [25]. Therefore, further studies of Sox2 should be carefully standardized and involve large sample sizes.

Nanog is also known as a prognostic factor associated with tumor progression and metastasis in breast cancer [10, 26]. Its prognostic significance was also reported in HER2-positive and triple-negative breast cancers [27, 28]. Although Nanog expression was associated with lymph node metastasis in this study, its expression was negatively correlated with other aggressive features of breast cancer (unlike Oct4 and Sox2) and did not show prognostic significance. As mentioned above, several analytical issues may be related to this discrepancy. Moreover, it may be associated with complex mechanism underling ESC transcription factor expression network [29]. Apostolou et al. identified some important genes affecting the expression of ESC transcription factors; these included thioredoxin-related transmembrane protein 2 (TMX2), family with sequence similarity 155, member B (Fam155B), and DEAD (Asp-Glu-Ala-Asp) box polypeptide 49 (DDX49). Knocking down DDX49 led to very low levels of Sox2 and Oct3/4, in parallel with an increase in Nanog level [30]. These results can be interpreted as evidence of the existence of an unknown network involving mutual regulation of the expression of the ESC transcription factors in breast cancer.

Bmi1 and Klf4 were negatively related to aggressive tumor characteristics and highly expressed in hormone receptor-positive tumors – the opposite findings to those for Oct4 and Sox2. In agreement with our data, Wang et al. have suggested that ERα binds to the promoter region of the *BMI1* gene and activates Bmi1 expression at the transcriptional level. Moreover, down-regulation of Bmi1 caused aberrant expression of p16^INK4a^, eventually leading to a high Ki-67 proliferation index [31]. Bmi1 expression was also associated with favorable overall survival in a sample of 960 breast cancer patients [32], and high Klf4 expression was reported to be associated with longer disease-free survival and overall survival of breast cancer patients [33]. Klf4 inhibited the transcriptional activity of ER-α and so suppressed estrogen-dependent breast cancer cell growth [34]. Also, nuclear factor I-C overexpression induced the expression of Klf4 and E-cadherin and eventually suppressed EMT, cell migration, and the invasiveness of breast cancer cells [35]. However, neither Bmi1 nor Klf4 were associated with prognosis in the total patient group or the hormone receptor-specific subgroups in our results.

Although further studies of these ESC transcription factors are needed, only Oct4 has the potential to be a useful prognostic marker for breast cancer. We found that Oct4 was strongly associated with the aggressive features of breast cancer, the ALDH1 expression, tamoxifen resistance and poor clinical outcome in hormone receptor-positive breast cancer. We therefore suggest that Oct4 expression may be used as an indicator of tumor progression and response to tamoxifen in hormone receptor-positive breast cancer.

## MATERIALS AND METHODS

### Patients and tissue samples

The specimens used in this study were surgically resected at Seoul National University Bundang Hospital, from 2003 to 2011, and diagnosed as primary invasive breast cancer (IBC). We collected IBC cases by slide review after searching an electronic database of pathology reports. Cases receiving preoperative systemic chemotherapy or presenting with initial metastases were excluded, and samples that were well fixed and contained a sufficient number of tumor cells were selected. Eventually, 319 breast cancer samples were included in this study. All the patients were treated according to standard practice guidelines and have been followed up regularly. This study was approved by the Institutional Review Board of Seoul National University Bundang Hospital (protocol # protocol # B-1601/332–304) and informed consent was waived.

### Construction of tissue microarrays

Formalin-fixed paraffin-embedded blocks containing representative tumor sections of the 319 cases of IBC were chosen and made into tissue microarrays (2mm in diameter, three core) (SuperBioChips Laboratories, Seoul, South Korea) for robust immunohistochemical analysis of ECS transcription factors.

### Immunohistochemical analyses

Oct4-, Sox2-, Nanog-, Bmi1-, and Klf4-specific antibodies were used to identify the transcription factors of ESCs. Information about these antibodies is given in Supplementary Table 6. We performed immunohistochemistry on thin sections (4μm) of tissue microarray slides to examine the transcription factors of ESC, BCSC markers and EMT markers, after optimizing staining using positive and negative controls and serial dilutions. The sections were cut, dried, deparaffinized and rehydrated following standard procedures. After that, the samples were heat-pretreated using retrieval solution and stained with antibodies in a BenchMark XT autostainer (Ventana Medical Systems, Tucson, AZ) using an ultraView detection kit (Ventana Medical Systemc), or manually with an Envision detection kit (Dako, Carpinteria, CA). Double-immunostaining to detect CD44+/CD24- cells was performed with EnVision G|2 Doublestain System Rabbit/Mouse (DAB+/Permanent Red) (Dako) according to the manufacturer's instructions.

The expression of markers was evaluated based on the proportion of tumor cells stained and the intensity of staining. After considering the distribution of proportions of positive cells expressing ESC transcription factors, samples showing strong nuclear staining in 10% or more of the tumor cells were considered positive, while the cut-off value for Sox2 was set at 1% of tumor cells due to the rarity in its expression. For expression of BCSC markers and EMT markers, the same cutoff values were used as in a previous study [36]. Cases diagnosed as invasive lobular carcinoma were excluded for evaluation of E-cadherin.

### Definition of breast cancer molecular subtypes

The molecular subtypes of breast cancer were defined according to the St. Gallen Expert Consensus as follows: luminal A subtype (ER+ and/or PR+, HER2-, Ki-67 < 14%), luminal B subtype (ER+ and/or PR+, Ki−67 ≥ 14%; ER+ and/or PR+, HER2+), HER2+ subtype (ER−, PR−, and HER2+) and triple-negative subtype (ER−, PR−, and HER2−) [37]. Expression of these basic biomarkers was evaluated at the time of diagnosis, or during the study in cases of missing data. For the hormone receptor (ER and PR), 1% or greater of nuclear staining was considered positive. For HER2, 3+ by immunohistochemistry or by identification of gene amplification by fluorescence *in situ* hybridization, was considered positive.

### Statistical analysis

The Statistical Package for the Social Sciences (SPSS) version 19.0 for Windows (SPSS Inc., Chicago, IL, USA) was used for statistical analysis. We used the chi-square test or Fisher's exact test for assessing the association between the expression of ESC transcription factors and the clinicopathologic features of breast cancer. The associations of clinicopathologic variables and ESC transcription factors with disease-free survival were analyzed and verified using the log-rank test, and the results were presented as Kaplan–Meier survival curves. All factors correlated with disease-free survival in the univariate analysis were incorporated in a Cox proportional hazards regression model using a backward stepwise selection method. Hazard ratio (HR) and its 95% confidence interval (CI) were calculated for each variable. Differences were considered statistically significant at *p* < 0.05.

## SUPPLEMENTARY MATERIALS TABLES


