# Supplementary material for: Expression of embryonal stem cell transcription factors in breast cancer: Oct4 as an indicator for poor clinical outcome and tamoxifen resistance
Source: Oncotarget. 2017 Mar 31;8(22):36305–18. doi: 10.18632/oncotarget.16750 (PMC5482656; doi:10.18632/oncotarget.16750)
Supplement: Supplementary file 1 [file oncotarget-08-36305-s001.pdf]

Expression of embryonal stem cell transcription factors in breast cancer: Oct4 as an indicator for poor clinical outcome and tamoxifen resistance

Supplementary Materials

Supplementary Table 1: Relationship between clinicopathologic characteristics of tumor and expression of Nanog, Bmi1, and Klf4 assessed by immunohistochemistry

Clinicopathologic Characteristics	Nanog		*p* value	Bmi1		*p* value	Klf4		*p* value
	Negative *N* (%)	Positive *N* (%)		Negative *N* (%)	Positive *N* (%)		Negative *N* (%)	Positive *N* (%)	
T stage			0.538			0.698			0.635
T1	132 (52.0)	31 (47.7)		79 (50.0)	84 (52.2)		136 (51.7)	27 (48.2)	
T2-T4	122 (48.0)	34 (52.3)		79 (50.0)	77 (47.8)		127 (48.3)	29 (51.8)	
N stage			0.009			0.447			0.527
N0	155 (61.0)	28 (43.1)		94 (59.5)	89 (55.3)		153 (58.2)	30 (53.6)	
N1-N3	99 (39.0)	37 (56.9)		64 (40.5)	72 (44.7)		110 (41.8)	26 (46.4)	
Histologic grade			0.015			0.002			0.022
I	35 (13.8)	10 (15.4)		14 (8.9)	31 (19.3)		38 (14.4)	7 (12.5)	
II	66 (26.0)	28 (43.1)		40 (25.3)	54 (33.5)		69 (26.2)	25 (44.6)	
III	153 (60.2)	27 (41.5)		104 (65.8)	76 (47.2)		156 (59.3)	24 (42.9)	
LVI			0.530			0.156			0.852
Absent	140 (55.1)	33 (50.8)		92 (58.2)	81 (50.3)		142 (54.0)	31 (55.4)	
Present	114 (44.9)	32 (49.2)		66 (41.8)	80 (49.7)		121 (46.0)	25 (44.6)	
P53 overexpression			0.072			0.040			0.048
Negative	170 (66.9)	51 (78.5)		101 (63.9)	120 (74.5)		176 (66.9)	45 (80.4)	
Positive	84 (33.1)	14 (21.5)		57 (36.1)	41 (25.5)		87 (33.1)	11 (19.6)	
Ki-67 index			0.166			0.001			0.168
< 20%	128 (50.4)	39 (60.0)		68 (43.0)	99 (61.5)		133 (50.6)	34 (60.7)	
≥ 20%	126 (49.6)	26 (40.0)		90 (57.0)	62 (38.5)		130 (49.4)	22 (39.3)	
ER			0.007			<0.001			0.009
Negative	87 (34.3)	11 (16.9)		70 (44.3)	28 (17.4)		89 (33.8)	9 (16.1)	
Positive	167 (65.7)	54 (83.1)		88 (55.7)	133 (82.6)		174 (66.2)	47 (83.9)	
PR			0.017			0.003			0.028
Negative	124 (48.8)	21 (32.3)		85 (53.8)	60 (37.3)		127 (48.3)	18 (32.1)	
Positive	130 (51.2)	44 (67.7)		73 (46.2)	101 (62.7)		136 (51.7)	38 (67.9)	
HER2			0.038			0.077			0.680
Negative	183 (72.0)	55 (84.6)		111 (70.3)	127 (78.9)		195 (74.1)	43 (76.8)	
Positive	71 (28.0)	10 (15.4)		47 (29.7)	34 (21.1)		68 (25.9)	13 (23.2)	

P values were calculated by chi-square test or Fisher's exact test.
LVI, lymphovascular invasion; ER, estrogen receptor; PR, progesterone receptor; HER2, human epidermal growth factor receptor 2.

Supplementary Table 2: Expression of Nanog, Bmi1, and Klf4 in relation to BCSC phenotypes and EMT markers

Markers	Nanog		*p* value	Bmi1		*p* value	Klf4		*p* value
	Negative *N* (%)	Positive *N* (%)		Negative *N* (%)	Positive *N* (%)		Negative *N* (%)	Positive *N* (%)	
CD44(+)CD24(−)			0.086			0.344			0.584
< 10%	126 (49.6)	40 (61.5)		78 (49.4)	88 (54.7)		135 (51.3)	31 (55.4)	
≥ 10%	128 (50.4)	25 (38.5)		80 (50.6)	73 (45.3)		128 (48.7)	25 (44.6)	
ALDH1			0.050			0.049			0.008
< 10%	226 (89.0)	63 (96.9)		138 (87.3)	151 (93.8)		233 (88.6)	56 (100.0)	
≥ 10%	28 (11.0)	2 (3.1)		20 (12.7)	10 (6.2)		30 (11.4)	0 (0.0)	
Vimentin			0.010			0.136			0.350
< 10%	210 (82.7)	62 (95.4)		130 (82.3)	142 (88.2)		222 (84.4)	50 (89.3)	
≥ 10%	44 (17.3)	3 (4.6)		28 (17.7)	19 (11.8)		41 (15.6)	6 (10.7)	
E-cadherin loss*			0.002			0.079			0.005
< 50%	169 (67.9)	57 (87.7)		106 (67.5)	120 (76.4)		178 (68.7)	48 (87.3)	
≥ 50%	80 (32.1)	8 (12.3)		51 (32.5)	37 (23.6)		81 (31.3)	7 (12.7)	

P values were calculated by chi-square test or Fisher's exact test.
ALDH1, aldehyde dehydrogenase 1
* Invasive lobular carcinoma cases were excluded.

Supplementary Table 3: Relationship between clinicopathologic characteristics of tumor and expression of Nanog, Bmi1, and Klf4 in the hormone receptor-positive subgroup

Clinicopathologic Characteristics	Nanog		*p* value	Bmi1		*p* value	Klf4		*p* value
	Negative *N* (%)	Positive *N* (%)		Negative *N* (%)	Positive *N* (%)		Negative *N* (%)	Positive *N* (%)	
T stage			0.464			0.408			0.598
T1	92 (54.8)	27 (49.1)		45 (50.0)	74 (55.6)		95 (54.3)	24 (50.0)	
T2-T4	76 (45.2)	28 (50.9)		45 (50.0)	59 (44.4)		80 (45.7)	24 (50.0)	
N stage			0.006			0.386			0.504
N0	100 (59.5)	21 (38.2)		52 (57.8)	69 (51.9)		97 (55.4)	24 (50.0)	
N1-N3	68 (40.5)	34 (61.8)		38 (42.2)	64 (48.1)		78 (44.6)	24 (50.0)	
Histologic grade			0.166			0.325			0.093
I	34 (20.2)	10 (18.2)		14 (15.6)	30 (22.6)		37 (21.1)	7 (14.6)	
II	59 (35.1)	27 (49.1)		34 (37.8)	52 (39.1)		61 (34.9)	25 (52.1)	
III	75 (44.6)	18 (32.7)		42 (46.7)	51 (38.3)		77 (44.0)	16 (33.3)	
LVI			0.463			0.210			0.936
Absent	89 (53.0)	26 (47.3)		51 (56.7)	64 (48.1)		90 (51.4)	25 (52.1)	
Present	79 (47.0)	29 (52.7)		39 (43.3)	69 (51.9)		85 (48.6)	23 (47.9)	
P53 overexpression			0.606			0.849			0.116
Negative	132 (78.6)	45 (81.8)		72 (80.0)	105 (78.9)		135 (77.1)	42 (87.5)	
Positive	36 (21.4)	10 (18.2)		18 (20.0)	28 (21.1)		40 (22.9)	6 (12.5)	
Ki-67			0.795			0.645			0.898
< 20%	116 (69.0)	39 (70.9)		61 (67.8)	94 (70.7)		122 (69.7)	33 (68.8)	
≥ 20%	52 (31.0)	16 (29.1)		29 (32.2)	39 (29.3)		53 (30.3)	15 (31.3)	
HER2			0.212			0.609			0.941
Negative	134 (79.8)	48 (87.3)		72 (80.0)	110 (82.7)		143 (81.7)	39 (81.3)	
Positive	34 (20.2)	7 (12.7)		18 (20.0)	23 (17.3)		32 (18.3)	9 (18.8)	
CD44(+)CD24(−)			0.768			0.883			0.737
< 10%	97 (57.7)	33 (60.0)		53 (58.9)	77 (57.9)		101 (57.7)	29 (60.4)	
≥ 10%	71 (42.3)	22 (40.0)		37 (41.1)	56 (42.1)		74 (42.3)	19 (39.6)	
ALDH1			0.198			0.444			0.351
< 10%	161 (95.8)	55 (100.0)		86 (95.6)	130 (97.7)		168 (96.0)	48 (100.0)	
≥10 %	7 (4.2)	0 (0.0)		4 (4.4)	3 (2.3)		7 (4.0)	0 (0.0)	
Vimentin			0.194			0.307			0.739
< 10%	156 (92.9)	54 (98.2)		83 (92.2)	127 (95.5)		164 (93.7)	46 (95.8)	
≥ 10%	12 (7.1)	1 (1.8)		7 (7.8)	6 (4.5)		11 (6.3)	2 (4.2)	
E-cadherin loss*			0.012			0.453			0.047
< 50%	122 (74.8)	50 (90.9)		68 (76.4)	104 (80.6)		130 (76.0)	42 (89.4)	
≥ 50%	41 (25.2)	5 (9.1)		21 (23.6)	25 (19.4)		41 (24.0)	5 (10.6)	

P values were calculated by chi-square test or Fisher's exact test.
*Invasive lobular carcinoma cases were excluded.
LVI, lymphovascular invasion; HER2, human epidermal growth factor receptor 2; ALDH1, aldehyde dehydrogenase 1.

Supplementary Table 4: Relationship between clinicopathologic characteristics of tumor and expression of Nanog, Bmi1, and Klf4 in the hormone receptor-negative subgroup

Clinicopathologic Characteristics	Nanog		*p* value	Bmi1		*p* value	Klf4		*p* value
	Negative *N* (%)	Positive *N* (%)		Negative *N* (%)	Positive *N* (%)		Negative *N* (%)	Positive *N* (%)	
T stage			0.750			0.202			0.723
T1	40 (46.5)	4 (40.0)		34 (50.0)	10 (35.7)		41 (46.6)	3 (37.5)	
T2-T4	46 (53.5)	6 (60.0)		34 (50.0)	18 (64.3)		47 (53.4)	5 (62.5)	
N stage			1.000			0.368			0.708
N0	55 (64.0)	7 (70.0)		42 (61.8)	20 (71.4)		56 (63.6)	6 (75.0)	
N1-N3	31 (36.0)	3 (30.0)		26 (38.2)	8 (28.6)		32 (36.4)	2 (25.0)	
Histologic grade			1.000			0.454			1.000
I	1 (1.2)	0 (0.0)		0 (0.0)	1 (3.6)		1 (1.1)	0 (0.0)	
II	7 (8.1)	1 (10.0)		6 (8.8)	2 (7.1)		8 (9.1)	0 (0.0)	
III	78 (90.7)	9 (90.0)		62 (91.2)	25 (89.3)		79 (89.8)	8 (100.0)	
LVI			0.735			0.969			0.472
Absent	51 (59.3)	7 (70.0)		41 (60.3)	17 (60.7)		52 (59.1)	6 (75.0)	
Present	35 (40.7)	3 (30.0)		27 (39.7)	11 (39.3)		36 (40.9)	2 (25.0)	
P53 overexpression			0.505			0.329			0.723
Negative	38 (44.2)	6 (60.0)		29 (42.6)	15 (53.6)		41 (46.6)	3 (37.5)	
Positive	48 (55.8)	4 (40.0)		39 (57.4)	13 (46.4)		47 (53.4)	5 (62.5)	
Ki-67			0.598			0.289			1.000
< 20%	11 (12.8)	0 (0.0)		6 (8.8)	5 (17.9)		10 (11.4)	1 (12.5)	
≥20 %	75 (87.2)	10 (100.0)		62 (91.2)	23 (82.1)		78 (88.6)	7 (87.5)	
HER2			0.514			0.761			0.715
Negative	49 (57.0)	7 (70.0)		39 (57.4)	17 (60.7)		52 (59.1)	4 (50.0)	
Positive	37 (43.0)	3 (30.0)		29 (42.6)	11 (39.3)		36 (40.9)	4 (50.0)	
CD44(+)CD24(−)			0.037			0.817			0.706
< 10%	29 (33.7)	7 (70.0)		25 (36.8)	11 (39.3)		34 (38.6)	2 (25.0)	
≥10 %	57 (66.3)	3 (30.0)		43 (63.2)	17 (60.7)		54 (61.4)	6 (75.0)	
ALDH1			1.000			0.878			0.192
< 10%	65 (75.6)	8 (80.0)		52 (76.5)	21 (75.0)		65 (73.9)	8 (100.0)	
≥10 %	21 (24.4)	2 (20.0)		16 (23.5)	7 (25.0)		23 (26.1)	0 (0.0)	
Vimentin			0.486			0.148			0.448
< 10%	54 (62.8)	8 (80.0)		47 (69.1)	15 (53.6)		58 (65.9)	4 (50.0)	
≥ 10%	32 (37.2)	2 (20.0)		21 (30.9)	13 (46.4)		30 (34.1)	4 (50.0)	
E-cadherin loss			0.505			0.910			0.459
< 50%	47 (54.7)	7 (70.0)		38 (55.9)	16 (57.1)		48 (54.5)	6 (75.0)	
≥ 50 %	39 (45.3)	3 (30.0)		30 (44.1)	12 (42.9)		40 (45.5)	2 (25.0)	

P values were calculated by chi-square test or Fisher's exact test.
LVI, lymphovascular invasion; HER2, human epidermal growth factor receptor 2; ALDH1, aldehyde dehydrogenase 1.

Supplementary Table 5: Kaplan-Meier survival analysis for disease-free survival

Group	Variable	*p* value
Whole study group	T stage (T1 vs. T2–4)	0.139
	N stage (N0 vs. N1–3)	0.017
	Histologic grade (I vs. II & III)	0.075
	LVI	0.009
	P53 overexpression	0.153
	Ki-67 index (< 20% vs. ≥ 20%)	0.600
	ER	0.753
	PR	0.538
	HER2 amplification	0.191
	CD44(+)CD24(–)	0.348
	ALDH1	0.272
	Vimentin	0.593
	E-cadherin loss	0.150
	Oct4	0.017
	Sox2	0.165
	Nanog	0.730
	Bmi1	0.495
	Klf4	0.243
	Adjuvant chemotherapy	0.266
	Adjuvant radiotherapy	0.829
	Adjuvant hormone therapy	0.625
HR-positive group	T stage (T1 vs. T2–4)	0.036
	N stage (N0 vs. N1–3)	0.007
	Histologic grade (I vs. II & III)	0.060
	LVI	0.087
	P53 overexpression	0.567
	Ki-67 index (< 20% vs. ≥ 20%)	0.706
	HER2 amplification	0.328
	CD44(+)CD24(–)	0.109
	ALDH1	0.395
	Vimentin	0.961
	E-cadherin loss	0.190
	Oct4	<0.001
	Sox2	0.384
	Nanog	0.416
	Bmi1	0.143
	Klf4	0.356
	Adjuvant chemotherapy	0.378
	Adjuvant radiotherapy	0.846
	Adjuvant hormone therapy	0.757

P values were calculated by log-rank test.
LVI, lymphovascular invasion; ER, estrogen receptor; PR, progesterone receptor; HER2, human epidermal growth factor receptor 2; ALDH1, aldehyde dehydrogenase 1; HR, hormone receptor.

Supplementary Table 6: Antibodies and staining conditions

Marker	Host	Clone	Dilution	Localization	Company
Oct4	Rabbit polyclonal	–	1:50	Nucleus	Abcam
Sox2	Rabbit monoclonal	SP76	Ready to use	Nucleus	Ventana
Nanog	Rabbit polyclonal	–	1:50	Nucleus	Abcam
Bmi1	Rabbit monoclonal	EPR3745(2)	1:50	Nucleus	Abcam,
Klf4	Mouse monoclonal	1E6	1:50	Nucleus	LifeSpan BioSciences
CD44	Mouse monoclonal	156-3C11	1:1000	Membrane	Abcam
CD24	Mouse monoclonal	SN3b	1:100	Membrane & cytoplasm	Thermo Scientific
ALDH1	Mouse monoclonal	44/ALDH	1:100	Cytoplasm	BD Bioscience
Vimentin	Mouse monoclonal	V9	1:1200	Cytoplasm	DAKO
E-cadherin	Mouse monoclonal	NCH-38	1:100	Membrane	DAKO

ALDH1, aldehyde dehydrogenase 1.
